# Exercise-Induced Muscle Damage and Protein Intake: A Bibliometric and Visual Analysis

**DOI:** 10.3390/nu14204288

**Published:** 2022-10-14

**Authors:** Fei Xu, Jinshu Zeng, Xuan Liu, Jiaming Lai, Jing Xu

**Affiliations:** 1 School of Physical Education, Hangzhou Normal University, Hangzhou 311121, China; 2 Division of Library and Information Services, Hangzhou Normal University, Hangzhou 311121, China; 3 San Diego Jewish Academy, San Diego, CA 92130, USA

**Keywords:** delayed-onset muscle soreness, nutritional strategy, protein intake, performance, eccentric exercise, muscle damage, inflammation, bibliometric analysis

## Abstract

Numerous studies have covered exercise-induced muscle damage (EIMD) topics, ranging from nutritional strategies to recovery methods, but few attempts have adequately explored and analyzed large volumes of scientific output. The purpose of this study was to assess the scientific output and research activity regarding EIMD and protein intake by conducting a bibliometric and visual analysis. Relevant publications from 1975–2022 were retrieved from the Web of Science Core Collection database. Quantitative and qualitative variables were collected, including the number of publications and citations, H-indexes, journals of citation reports, co-authorship, co-citation, and the co-occurrence of keywords. There were 351 total publications, with the number of annual publications steadily increasing. The United States has the highest total number of publications (26.21% of total publications, centrality 0.44). Institutional cooperation is mostly geographically limited, with few transnational cooperation links. EIMD and protein intake research is concentrated in high-quality journals in the disciplines of Sport Science, Physiology, Nutrition, and Biochemistry & Molecular Biology. The top ten journals in the number of publications are mostly high-quality printed journals, and the top ten journals in centrality have an average impact factor of 13.845. The findings of the co-citation clusters and major keyword co-occurrence reveal that the most discussed research topics are “exercise mode”, “nutritional strategies”, “beneficial outcomes”, and “proposed mechanisms”. Finally, we identified the following research frontiers and research directions: developing a comprehensive understanding of new exercise or training models, nutritional strategies, and recovery techniques to alleviate EIMD symptoms and accelerate recovery; applying the concept of hormesis in EIMD to induce muscle hypertrophy; and investigating the underlying mechanisms of muscle fiber and membrane damage.

## 1. Introduction

Exercise-induced muscle damage (EIMD) is a phenomenon that occurs following novel or unaccustomed exercise, particularly if the exercise includes a high number of eccentric contractions [1]. The damage process is normally accompanied by a temporary decrease in muscle function (both muscle force and range of motion) [2,3], increased swelling of the involved muscle group [2], increased circulation of muscle-specific proteins [4], and delayed-onset muscle soreness (DOMS) [5]. Most of the symptoms and signs of EIMD are present immediately after the initial exercise bout and persist for up to 14 days [6]. These variables are also widely used to assess the extent of muscle damage, with DOMS being the most commonly assessed marker, despite the fact that the underlying mechanisms of its occurrence remain inconclusive [7,8].

Although the exact mechanisms causing EIMD remain unclear, the damage model can be simplified into the initial phase or primary damage that occurs during the exercise bout and the secondary damage phase that occurs after exercise and is associated with an inflammatory response [9]. Various preventive and therapeutic methods are used to attenuate EIMD [10]. Nutritional intervention, in particular, is essential for modulating oxidative stress and inflammation, both of which are thought to be contributing factors to EIMD but also important for the adaptive response to an exercise stimulus [11]. Given that the nutritional strategy focuses on maximizing exercise recovery, nutrients and functional foods with the potential to alleviate EIMD are investigated [6]. As a consequence, to balance recovery and adaptation, nutritional strategies for alleviating EIMD symptoms need to be modified depending on the main purpose of the session [6,12].

Despite numerous research contributions to EIMD, the exact mechanisms causing muscle damage, soreness, repair, and adaptation from eccentric exercise remain speculative. Furthermore, there is much to be revealed about nutrition, such as the underlying mechanisms of the effects of functional foods and their derivatives, as well as the long-term effectiveness of various nutritional interventions [6,12]. Numerous studies have been conducted on a wide range of topics, including nutritional strategies and recovery techniques and complex mechanisms involving the investigation of various immunological [1,6,10,13] and endocrine signaling molecules [13,14,15].

Uncertainty arises from the fact that EIMD is a complex area of study involving a variety of factors, such as fitness level, nutrition, genetics, sex, age, and familiarity with the exercise mode [13], raising the question of whether using supplements and functional foods to treat EIMD is the best practice. A variety of recovery and treatment strategies have been introduced to help alleviate the severity of EIMD and restore muscle function as quickly as possible. These include stretching, massage, cryotherapy, ultrasound, and electrical stimulation [1,5,10,13,16,17]. None of them, however, are fully recognized. Surprisingly, few attempts have adequately addressed the broad range of studies and thoroughly investigated the scientific output.

As a plausible method for qualitatively and quantitatively evaluating publications on a specific subject, bibliometric analysis, also known as scientometric analysis, is an optimal choice for exploring scientific knowledge domains and trends over time [18]. When applied to scientific research, it enables us to summarize large amounts of bibliometric data in order to present the state of knowledge and emerging trends of a research topic or research field over time. To the best of our knowledge, the use of bibliometric and systematic mapping is still in its early stages, and no specific bibliometric analyses on EIMD and protein intake have been performed [19,20]. As a result, using an appropriate scientometric method to reveal the current state, frontiers, and future research trends of this topic is critical. As a result, we sought to provide a broad overview perspective via bibliometric and visual analysis in order to gain a better understanding of EIMD and protein ingestion research as well as insights into future research trends.

## 2. Materials and Methods

The study protocol was informed by the concept of “research weaving” proposed by Nakagawa et al. (2019) [18], a new framework for the research synthesis of both evidence and influence, accounting for their trends over time. Research weaving combines the power of two methods: (1) bibliometrics, which shows how pieces of evidence are connected, revealing the structure and development of a scientific field, and (2) systematic mapping, which provides a snapshot of the current state of knowledge, identifying areas that require more research attention and those ready for full bibliometric synthesis. This framework summarizes and visualizes information on a collection of papers on any given topic [19,20,21].

### 2.1. Search Strategy and Data Collection

We obtained data from the Web of Science Core Collection (WoSCC) database on 4 June 2022. WoSCC has been widely used for the retrieval of scientific publications and for scientometric analyses [19,20]. To collect data comprehensively, we searched WoSCC from its inception to 2022 (1975–2022). The retrieval strategy was as follows: topics (title, keywords, abstract) = (“exercise-induced muscle damage” OR “EIMD” OR “delayed onset muscle soreness” OR “DOMS”) AND (“protein” OR “protein intake” OR “protein ingestion”); category = Science Citation Index Expanded (SCI-Expanded), Social Sciences Citation Index (SSCI), Arts & Humanities Citation Index (A&HCI), Conference Proceedings Citation Index Science (CPCI-S), Conference Proceedings Citation Index-Social Science & Humanities (CPCI-SSH), Emerging Sources Citation Index (ESCI), Current Chemical Reactions (CCR-Expanded), Index Copernicus (IC); document type = article, review, and conference reports; language = English. The search results from WoS were subsequently filtered to include peer-reviewed studies.

Our study included studies that met the following criteria: (1) EIMD/DOMS and protein ingestion/intake; (2) the effects of different exercise patterns on EIMD or DOMS (including muscle microstructure damage); (3) EIMD/DOMS-related myofibrillar/muscle fiber, muscle cell/muscle cell membrane/sarcolemma/cytoskeleton proteins and protein ingestion/intake; and (4) EIMD/DOMS and nutritional supplements. Studies were excluded if they met the following criteria: (1) incompatibility with the EIMD or DOMS and protein intake/ingestion topic; (2) the absence of muscle damage, muscle soreness, and protein ingestion as primary topics; and (3) an emphasis on EIMD/DOMS-related healthcare and philosophy and the absence of muscle and protein indicators; (4) publications in a language other than English, non-peer-reviewed research, and duplicate publications (including conference papers and journal articles); and (5) an original article or short communication that does not include a complete presentation of the experimental design or a review that does not include a detailed discussion and a clear conclusion. With the aforementioned criteria, we finally included 351 records (Table S1: Search strategy and results).

All records and references were downloaded and saved in plain text format for further analysis. To avoid bias from database updates, the literature search was completed on a single day. Full-text records were downloaded for further quantitative and qualitative analysis. Two authors independently conducted a literature search that strictly adhered to the inclusion and exclusion criteria, removing duplicate studies and extracting various outputs, such as research countries, institutions, authors, journals, keywords, and citations [20].

### 2.2. Measures

We assessed the quality of scientific information using the Journal Impact Factor (JIF) and quartile category data (Q) from JCR. The annual impact factor (IF) for a journal published by the Web of Science (WoS) JIF section is the average number of citations for each article published in a specific journal over the previous two years. InCites shows the top quartile of journals from multiple WoS research areas. When a research area is specified, the quartile for that journal and research area is displayed, indicating scientific “prestige”. Furthermore, the H-index was considered, which is defined as one researcher publishing *h* articles with at least h citations each. It is widely accepted as a metric for evaluating the scientific output (productivity), impact, and academic standing of an institution, journal, or researcher [22].

Co-citation reference networks. These networks allow researchers to estimate so-called hotspots [19,20] or units of measurement for authors, references, countries, institutions, or keywords that have significantly higher connections than others [21]. Technically, co-citation reference networks are based on the concept of co-citation networks, which are a method of visualizing highly cited and closely related publications. Co-citation networks are built by connecting two documents that are both cited by a third [23]. It detects critical transitions more accurately over time [18]. Co-citation networks enriched by thematic patterns of article citations, as used in this study, may highlight research frontiers and the scholarly impacts of the intellectual base.

Co-occurrence analysis. It is the counting of paired data within a collection unit, such as keywords. The majority of studies involved identifying keywords in the text, calculating the frequencies of keyword co-occurrences, and locating clusters of keywords in the network [19,20,24].

In relation to our objectives, (1) Author and co-authorship analysis reveals collaborative relationships between authors, which may assist researchers in better understanding current collaborations and identifying potential collaborators. (2) Co-citation analysis involves tracking pairs of studies that are cited together in the source references, illuminating a current research topic. The strength of co-citations assists researchers in identifying the intellectual foundation and research frontiers. (3) Keyword co-occurrence helps researchers identify research hotspots and trends, as well as inspire new research ideas by investigating the links between keywords in the literature.

Overall, research-weaving tools used in relation to co-citation reference networks and keyword co-occurrence included systematic mapping, intellectual structure, and collaboration networks [25]. Research-weaving tools used for the secondary objectives included performance analysis and collaborative (influence) network analysis (Table S2: Key concepts in bibliometrics and in network analysis).

### 2.3. Data Analysis and Software

We performed this bibliometric and visualization analysis with CiteSpace V.5.8.R3 (64-bit) software, an interactive visualization tool that combines bibliometric, information visualization, and Java data-mining algorithms [19,26]. CiteSpace was created to assist researchers in creating knowledge network maps, tracing scientific developments, and identifying emerging hotspots in a specific field [19,26]. In our study, we analyzed the publications, full references, and citations of the retrieved articles for countries/regions, institutions, and journals; additionally, co-authorship, co-citation, and keyword co-occurrence analyses are the three most important [19,20,26].

## 3. Results

### 3.1. Publication Outputs and Citations

**Figure 1:**
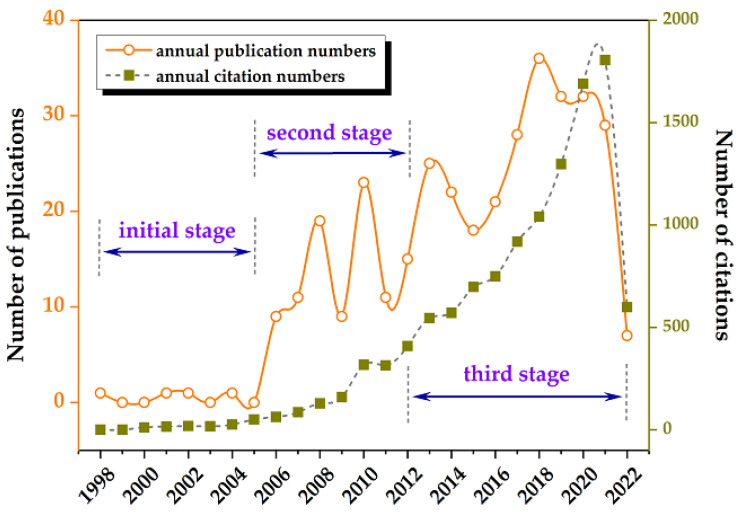
The number of publications and citations.

We retrieved results that showed the earliest study pertaining to this topic in WoSCC to be that by Pedersen et al. (1998) [27]. Therefore, this study was limited to data from 1998 to 2022. A total of 351 publications met the inclusion criteria and were used for subsequent analysis. According to Figure 1, the number of annual publications has steadily increased with some fluctuations, from 1 in 1998 to 29 in 2021, divided into three stages: the first (1998–2005), the second (2006–2012), and the third (2013–2016). There were initially only a few publications each year. In the second stage, there were typically no more than 20 annual publications. The number kept rising, with typically more than 20 publications in each year of the third stage. In addition, this corresponds to an increase in citations across all three stages. In 2021, there were 1805 citations for a total of 11,537.

### 3.2. Contributions of Countries and Institutions

**Figure 2:**
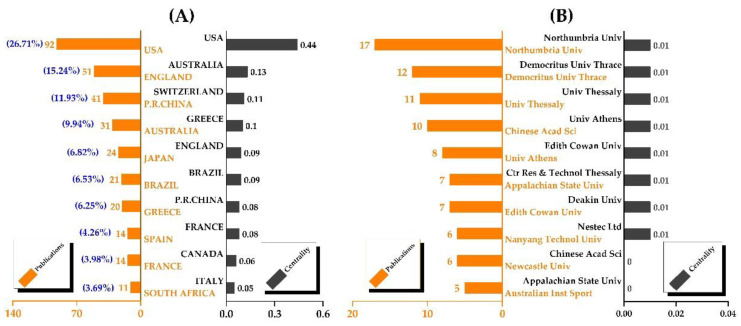
The contributions of countries and institutions. (**A**) The contributions of countries; (**B**) the contributions of institutions.

This bibliometric analysis involved 60 countries/regions and 359 institutions in total. The top ten countries dominated in the number of contributions, accounting for 90.88% of publications (Figure 2A). The United States had the most publications (92, 26.21%), followed by England (51, 14.53%), China (41, 11.68%), Australia (31, 8.83%), and Japan (24, 6.84%). The United States (0.44), Australia (0.13), Switzerland (0.11), and Greece are in descending order of centrality (0.1). Intriguingly, Switzerland had the third-highest centrality (0.11) but only three publications. Figure 2B shows that the United States’ institutions did not have a ranking advantage. On the other hand, universities in England (Northumbria University and Newcastle University), Greece (Democritus University of Thrace, University of Thessaly, University of Athens, and Center for Research and Technology, Thessaly), and Australia (Edith Cowan University, Australian Institute of Sport, and Deakin University) were more prominent. Surprisingly, none of the institutions had a high degree of centrality.

**Figure 3:**
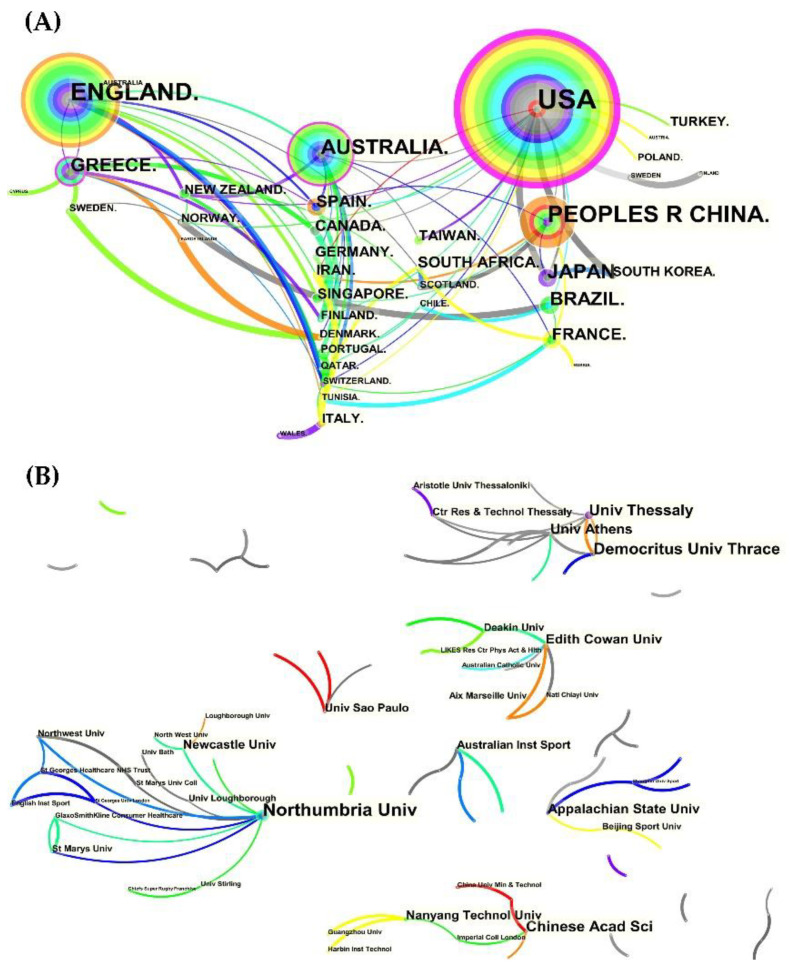
The collaboration network of countries and institutions. (**A**) The collaboration network of countries; (**B**) the collaboration network of institutions.

The collaboration network maps (Figure 3) show that the United States, England, and Australia had the largest nodes and the densest connectivity, indicating a high level of national cooperation. In comparison to Greece, Japan, and even smaller-node countries with greater international cooperation, China had a large node but few connections, indicating a lack of cooperation and connections. Furthermore, when we refined the results to the institutional level, we found that, while the US led at the national level, the UK and Greece were better represented in terms of authors and institutions. It should be noted, however, that institutional cooperation was mostly limited to the same country/region, with geographical constraints and few transnational cooperation links.

### 3.3. Author and Co-Cited Authors

**Figure 4:**
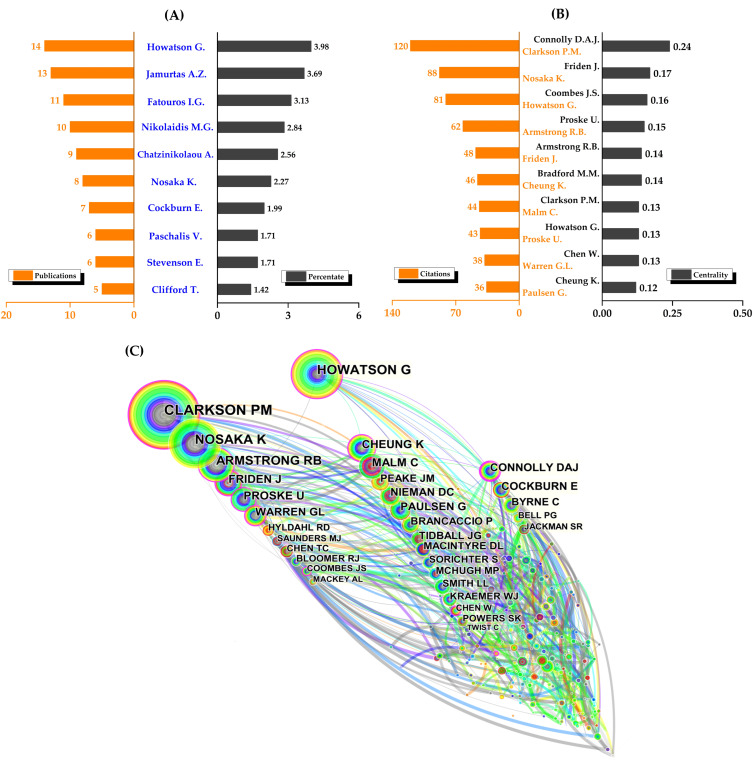
The top ten authors and co-cited authors and co-authorship networks. (**A**) Top ten authors; (**B**) top ten co-cited authors; and (**C**) co-authorship networks.

There were 590 authors who contributed to the total number of publications, with the top 10 authors having a total of 89 publications (Figure 4A). Only four authors had at least 10 publications, with Howatson having the most (14), followed by Jamurtas (13), Fatouros (11), and Nikolaidis (10). Among the top ten co-cited authors (Figure 4B), Clarkson was the only author in the top ten with more than 100 co-cited records (120), followed by Nosaka (88), Howatson (81), Armstrong (62), and Friden (48). Notably, Connolly had the highest centrality (0.24) with 35 co-citation counts, followed by Friden (0.17), Coombes (0.16), Proske (0.15), and Armstrong (0.14). Surprisingly, Bradford’s centrality was 0.14 with only two co-citations, but one of his earlier methodological studies on the rapid and sensitive quantification of protein utilization had been cited over 200,000 times in WoS [28]. 

In addition to the information provided above, Figure 4C visually and intriguingly illustrates the collaboration of co-cited authors.

### 3.4. Journals and Co-Cited Journals

**Figure 5:**
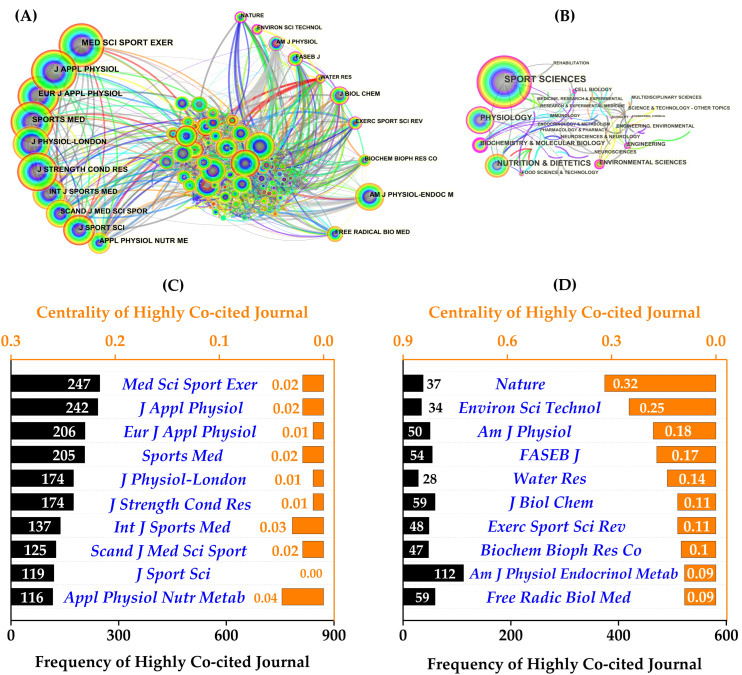
The visualization of the co-citation network and information on the top ten co-cited journals. (**A**) The network map of co-cited journal collaboration and (**B**) discipline categories; (**C**) the frequency and (**D**) centrality of publications in the top ten co-cited journals.

The results for the top ten most published journals (Table 1) and journal categories (Figure 5B) revealed that EIMD and protein intake research focused on the discipline categories of Sport Science, Physiology, Nutrition, and Biochemistry & Molecular Biology. Eur J Appl Physiol (IF = 3.346, H-index = 142) published the most references (20 publications, 5.68%), followed by Appl Physiol Nutr Metab (IF = 3.016, H-index = 95) with 16 publications (4.46%) and Med Sci Sports Exerc (IF = 6.289, H-index = 238) with 13 publications (3.69%). It is worth noting that the journals in Table 1 are all of high quality and are all ranked in the Q1/Q2 range in their research categories.

Interestingly, the collaborative network map and detailed information from the co-cited journals (Figure 5A) revealed that the top ten journals by frequency and centrality were completely different. Four journals accounted for over 200 co-citations each (Figure 5C): Med Sci Sports Exerc (247), J Appl Physiol (242), Eur J Appl Physiol (206), and Sports Med (205). In terms of centrality (Figure 5D), Nature ranked first (0.32), followed by Environ Sci Technol (0.25), Am J Physiol (0.18), and The FASEB J (0.17).

### 3.5. References and Co-Cited References

The top ten most-cited references were published from 1998 to 2017 (Table 2). Clarkson et al.’s work [1] received the most citations (840), propelling it to the top of the list, followed by Howatson et al. [10] (302), Friden et al. [29] (268), Ispirlidis et al. [30] (243), Peake et al. [31] (229), and Shimomura et al. [32] (213), all of which received more than 200 citations. Intriguingly, the top ten cited and co-cited references (2004–2019) barely overlapped (Table 2 and Table 3), with only Howatson et al.’s research (302 citations, centrality 0.23) [10] ranked second in both lists. Notably, Cockburn was the first author of two highly co-cited references [33,34] that ranked first (centrality 0.29) and third (0.20). Furthermore, Peake’s study [31] and Owens’ work [6] had the most citations (229) and the highest centrality (0.14) among references published in the last five years, respectively.

**Figure 6:**
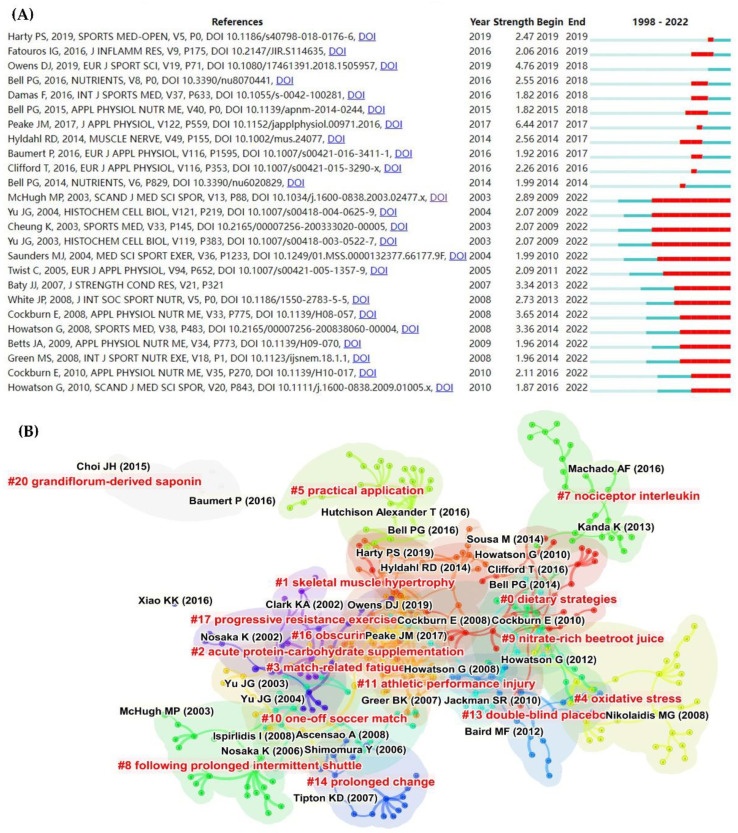
The visualization of the co-citation network and information on the top ten co-cited journals. (**A**) The network map of co-cited journal collaboration and (**B**) discipline categories. Black font indicates representative co-cited references; red font with # indicates cluster labels and cluster names of co-cited references [1,2,3,4,5,6,7,8,9,10,11,12,13,14,15,16,17,18,19,20,21,22,23,24,25,26,27,28,31,33,34,36,38,39,40,41,42,43,44,45,46,47,48,49,50,51,52,53,54,55,56,57,58,59,60,61,62,63,64,65,66,67].

CiteSpace identified the top 25 references with the strongest citation bursts (Figure 6A). We noticed an intriguing “stratification”: the first half of the literature is relatively new (2014–2019) but of short duration. Although the second half of the literature was published earlier (2003–2010), the citation bursts continue to date. Future research frontiers are thought to be fundamentally based on “references with citation bursts,” which show that the corresponding studies were frequently cited within a particular timeframe [20]. Therefore, mining the information in both parts of the literature is likely to yield significant results.

On the other hand, based on co-citation analysis, a total of 16 major clusters were recognized (Figure 6A). We divided these clusters into four categories: exercise modes (#3 match-related fatigue, #8 following prolonged intermittent shuttle, #10 one-off soccer match, #11 athletic performance injury, and #17 progressive resistance exercise), nutrition strategies (#0 dietary strategies, #2 acute protein–carbohydrate supplementation, #9 nitrate-rich beetroot juice, #20 grandiflorum-derived saponin, and #13 double-blind placebo), beneficial outcomes (#1 skeletal muscle hypertrophy, #5 practical application, and #14 prolonged change), and mechanisms (#4 oxidative stress, #7 nociceptor interleukin, and #16 obscurin).

### 3.6. Analysis of Keywords

**Figure 7:**
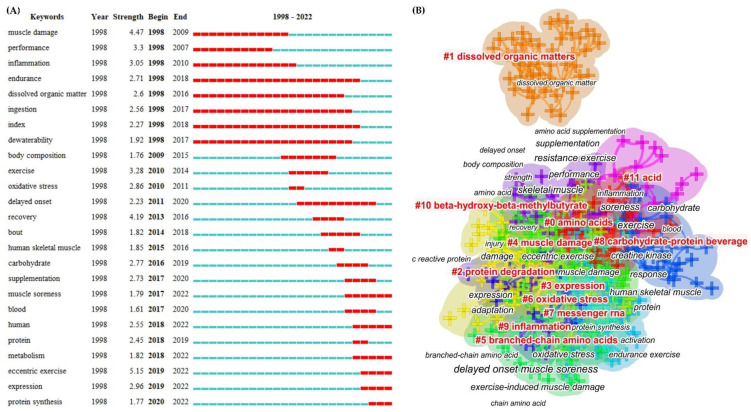
Keyword visualization of frequency and centrality, strongest citation bursts, and cluster mapping. (**A**) Top 25 keywords with the strongest citation bursts; (**B**) the keyword clustering map. Note: Begin, start time of keyword burst; End, end time of keyword burst. Legend: ▂ one year; ▃ burst year. Black font indicates representative co-cited references; red font with # indicates cluster labels and cluster names of co-cited references.

This study included 424 keywords in total. Figure 7A revealed that the only keyword used more than 100 times (113) was eccentric exercise, followed by skeletal muscle (78), muscle damage (75), performance (70), and recovery (51). The top five high-centrality keywords were, in order, performance (0.28), exercise (0.23), skeletal muscle (0.22), delayed-onset muscle soreness (0.21), and dissolved organic matter (0.2). Moreover, as shown in Figure 7A, eccentric exercise was the strongest burst keyword (strength 5.15) and emerged in 2019, followed by muscle damage (4.47, 1998), recovery (4.19, 2013), performance (3.30, 1998), and exercise (3.28, 2010).

A total of 12 major keyword clusters were identified as highly compatible with the purpose of this study (Figure 7B). We classified all of the clusters into two categories: nutritional strategies (#0 amino acids, #1 dissolved organic matter, #2 protein degradation, #5 branched-chain amino acids, #8 carbohydrate–protein beverage, #10 beta-hydroxy-beta-methylbutyrate, and #11 acid) and proposed mechanisms (#4 muscle damage, #3 expression, #6 oxidative stress, #7 messenger RNA, and #9 inflammation).

## 4. Discussion

### 4.1. Research Sources

From 1998 to 2022, the total number of publications on protein intake and EIMD steadily increased, albeit with some fluctuations, and reached 351 publications. The top ten authors dominated in the number of contributions, making up 90.88% of publications (319/351) on this topic, despite having authors from 60 different countries. The United States led in the number of publications (92, 26.21%), centrality (0.44), and international collaboration with peers, followed by England (51, 14.53%) and China (41, 11.68%). This research area was supported by 590 authors from 359 institutions in various countries, but only 4 from two countries (Howatson, UK; Jamurtas, Fatouros, and Nikolaidis, Greece) had published more than ten articles. Moreover, after Northumbria University (UK), which had the most publications, Greek institutions ranked second and third. Therefore, the United States’ national-level advantage does not appear to be supported by authors and institutions.

We also discovered that many eminent scholars hold key positions in co-authorship networks (Figure 4C). Outstanding scholars such as Saltin [68,69] and Bangsbo [70,71,72] in Denmark, Fridén [73] and Malm [74,75] in Sweden, Vihko and Salminen [76,77] in Finland, and Armstrong’s research group (including Warren and Schwane et al.) [78,79,80] in the United States have made significant contributions to the field of muscle injuries and muscle damage. The significant contributions of these outstanding professors have served as the foundation for ongoing breakthroughs in this field. Carl Foster sincerely praised Professor Saltin as “*A Role Model for More Than a Generation of Scientists,*” and those illustrious scholars deserve to be admired.

According to our findings, the most important research categories for protein-related EIMD research were Sport Science, Physiology, Nutrition, and Biochemistry & Molecular Biology. Eur J Appl Physiol (20 publications), Appl Physiol Nutr Metab (16), and Med Sci Sports Exerc (13) had the most publications, while J Appl Physiol (240), Med Sci Sports Exerc (238), and Eur J Appl Physiol (142) had the highest H-index. Furthermore, the top ten co-citation frequency journals are all established journals in the Sport Science and Physiology discipline category (all print journals, IF ranging from 2.997 to 11.928, average 5.110), and the majority of high-centrality journals have relatively high impact factors (ranging from 3.322 to 69.504, average 13.845). Despite the fact that the top ten journals by frequency and centrality were completely different, it is difficult to pinpoint which journal or journals are more important in this field. Our findings are useful for researcher communication and manuscript submission to appropriate potential journals. However, getting published in these high-quality journals is challenging.

### 4.2. Research Topics

The network of co-cited journals and co-citations is typically used to analyze a specific intellectual link and provide the following important information, which is commonly regarded as the foundation of a specific research field, thereby facilitating knowledge-domain exploration [19,20]. We focused on the co-citation clustering results (Figure 6) and combined the most cited and co-cited references (Table 2 and Table 3) to suggest the following research topics:

- Exercise modes. Exercise modes that can result in EIMD and/or DOMS include long-distance running [81], resistance exercise [82], high-intensity intermittent exercise [83], and downhill running [84]. Eccentric contractions cause more severe symptoms than isolated or concentric contractions [1]. Muscle-damaging exercise modes are also classified as aerobic exercise and strength training [13]. Interestingly, both our clustering results (#10 one-off soccer match, #3 match-related fatigue, #11 athletic performance injury, and #17 progressive resistance exercise) and recent studies revealed that the effect of specific sports (e.g., soccer [85] and basketball [86]) on muscle damage is a research focus. This implies that specific sports with greater ecological validity are receiving increased attention. We believe that improving the ecological validity of studies for better application can provide useful insights into how to avoid cumulative fatigue and overtraining, as well as lower the risk of injury. While the preceding studies [85,86] examined muscle damage, their primary purpose was to review or investigate the effects of a one-off match (combined with supplementation) on athletic performance. From this perspective, the reduced risk of injury and improved human performance in real-world settings [85,86,87] broaden the application scenarios of “exercise modes”. Another application is performing an unusual eccentric exercise that triggers the so-called “repetitive bout effect (RBE)”, which has been shown to be a protective adaptive response that significantly reduces EIMD [88]. Therefore, as an important research topic, “exercise mode” has evolved from human- and animal-tested exercise protocols to the current training and intervention model.
- Nutritional strategies and recovery techniques. Co-citation clustering (#0, #2, #9, #13, and #20), as well as several high-cited and high-co-cited references [33,34,36,89], and keyword clustering (Figure 7B) all pointed to “nutritional strategies” as one of the most dominant research topics in the field of EIMD and protein intake. Not only is “diet strategies” (#0) the top-ranked co-citation cluster, but it is also widely accepted that optimal nutritional supplementation is essential for muscle repair and regeneration [90].
- Specifically, branched-chain amino acids (BCAA) are effective supplements for reducing muscle damage and accelerating recovery after exercise [32,35]. *β*-Hydroxy-*β*-methylbutyrate (HMB), a BCAA metabolite, has gained popularity as a human nutritional supplement, particularly among strength athletes [91], by preventing muscle protein degradation and muscle damage during intense training [10]. Another promising strategy for reducing muscle damage is the combination of carbohydrates and protein (e.g., carbohydrate–protein beverages) [33,34]. Furthermore, in the context of nutritional strategies, the parallel to nutritional supplements is diet and food. The importance of diet and food is supported by the top ten co-cited references (three studies using milk or Montmorency cherry) and co-citation clusters [33,34,36,89]. Diet and food containing various ingredients (clusters #9 nitrate-rich beetroot juice and #20 grandiflorum-derived saponin) have also been studied to alleviate EIMD [66,92]. Overall, diet and food are a more practical and secure alternative to supplements for athletes to prevent unintentional doping [93]. It is also worth noting that when conducting a clinical trial (e.g., nutritional changes or massage), it has become standard practice to use a randomized, double-blind, and placebo study design whenever possible to avoid expectations (bias) of main and subject/participant effects. Therefore, double-blind placebo was identified as a significant cluster (#13) for this research topic. Finally, skeletal muscle inflammation and soreness are frequently treated with non-nutritive recovery techniques, such as massage and cold-water immersion [16,17]. Although physical recovery techniques were not included in the clustering of co-cited references and keywords, two highly cited studies [16,17] were among the top ten cited references. Combining the evidence presented above and considering the practical applications of physical recovery techniques in the field of sports and fitness (likely accompanied by EIMD or DOMS), it is reasonable to conclude that non-nutrition treatments are a promising research topic. Intriguingly, the potential mechanisms by which physical recovery techniques affect EIMD or DOMS, as well as whether they interact with nutritional supplements, need to be investigated further. To summarize, gaining a comprehensive understanding of nutritional strategies to alleviate EIMD symptoms and accelerate recovery [6,10] is the predominant research objective.
- Beneficial outcomes. As previously stated, the EIMD- and DOMS-related “exercise mode” and “nutritional strategies” are becoming more application-oriented. This implies that “beneficial outcomes” will inevitably receive more attention, as evidenced by cluster #5 (practical application), which is provided by Peake et al.’s review [31]. Cluster #1 (skeletal muscle hypertrophy) demonstrates that muscle inflammation and increased protein turnover during EIMD recovery are required for long-term hypertrophic adaptations [14]. Peake et al.’s review concluded that EIMD and the subsequent inflammatory responses are unavoidable because both are thought to be essential components of muscle repair [31]. Furthermore, as evidenced by the top-cited and top co-cited references (Table 2 and Table 3), numerous application-based studies on training interventions and nutritional supplements have highlighted their beneficial outcomes. Measures of exercise performance (muscle strength, vertical jump, and speed), muscle function and muscle cell morphology (muscle cell and subcellular disruptions, muscle swelling, and cytoskeletal muscle fiber components), the inflammatory response (leukocyte count, C-reactive protein, cytokines, and interleukin-6), enzymes (creatine kinase and lactate dehydrogenase), and hormones/metabolites are the main components included (cortisol, testosterone, thiobarbituric acid-reactive substances, protein carbonyls, and uric acid). Given the previous findings, the role of EIMD in muscle function and hypertrophy is an especially intriguing research hotspot.
- Taking all of the above outcomes into account, the role of EIMD in muscle function and hypertrophy is an intriguing research hotspot. Muscle hypertrophy, for example, can benefit the elderly’s health by preventing the onset of sarcopenia and (or) muscle atrophy [94]. Therefore, this research topic is supported by a large number of beneficial outcomes. However, transferring the beneficial effects from the laboratory to the clinic remains a challenge.
- Proposed mechanisms. Exploring the underlying mechanisms of EIMD or DOMS has always been regarded as an important research topic. It is widely accepted that the complex damage model can be divided into two phases: the initial phase, or primary damage, that results from the mechanical and metabolic stress caused by an exercise bout, and secondary damage that propagates tissue damage via processes associated with the inflammatory response [9]. Numerous studies, as evidenced by the clusters of co-citations and keywords (Figure 6 and Figure 7), concentrated on oxidative stress and inflammation, which are thought to be EIMD contributing factors but are also required for recovery and adaptation processes [6,11]. It is also important to recognize muscle protein functions (co-citation cluster #16 obscurin) and pro-inflammatory cytokine gene expression (cluster #7 messenger RNA and keyword cluster #7 nociceptor interleukin). Finally, assessing the efficacy of the proposed EIMD-associated-protein mechanisms assists researchers in determining the precise effect of various nutritional and therapeutic strategies on EIMD reduction and recovery [4,13].

### 4.3. Frontiers and Future Directions

In general, research frontiers are inferred by analyzing the evolution of keyword bursts and end years [26]. However, we revealed that using only high-frequency keyword data is insufficient, potentially ignoring some high-quality research [19,20]. As a result, we combined burst keywords and keyword clusters (Figure 7) with a co-cited reference timeline map (Figure 6). The frontiers can be categorized as follows.

- The foundation of muscle hypertrophy and protein supplements. In recent years, investigating whether or not EIMD is related to muscle hypertrophy appears to be cutting-edge, as the frontier keywords “protein synthesis” and “eccentric exercise” have an association with muscle hypertrophy. Although muscle hypertrophy can occur in the absence of muscle damage [95,96], increased exercise-induced reactive oxygen species (ROS) and inflammatory responses are more commonly associated with muscle hypertrophy and are attracting researchers’ attention. Muscle adaptation necessitates low-to-moderate-exercise-induced ROS production; if it exceeds a certain threshold, not only do the physiological benefits diminish, but muscle damage worsens [97]. Although post-exercise oxidative stress and inflammation are important for both recovery and adaptation [11], long-term nutritional interventions that rely too heavily on these two factors have not resulted in the expected improvement in muscle adaptation [6]. Moreover, the trade-off between recovery and adaptation varies depending on the primary goal of the session. To optimize the “beneficial outcomes”, for example, a periodized approach to nutrition should be fully considered to adequately support exercise/training in order to achieve a balance between the potential for recovery and adaptation [6,12]. This might be partially supported by the concept of exercise-induced hormesis theory, which purports that biological systems respond with a bell-shaped curve [97,98]. Furthermore, the first half of the “stratification” of burst references (2014–2019) in Figure 6A reflects and corroborates this. Therefore, it is likely to become a research frontier that applies the concept of hormesis of nutritional supplementation to various training purposes, training phases, fitness levels, genders, and ages and then applies these findings in real-world scenarios.
- In practice, however, a single theory and/or hypothesis has limited explanatory power when the cause–effect relationship is not proven. More research is needed to confirm that there is a link between muscle damage and hypertrophy [14] and to investigate the potential dose–response relationship. The optimal level for determining muscle damage caused by hypertrophy can be determined in this direction. Thus, we believe that “exercise modes” and “nutritional strategies” are important in activating antioxidants, DNA repair, and protein-degrading enzymes. It is more than just muscle recovery and adaptation and includes, perhaps more subtly, oxidative stress-related diseases and, more broadly, aging.
- Potential mechanisms of muscle fiber and muscle membrane damage. Applied research is frequently supported by basic science. Aside from the aforementioned applied research, the evidence presented below has a common but easily overlooked theme: the “stratification” of citation burst references (2003–2010, Figure 6B), which have been continuously followed and cited until now. Influential basic studies can be found in high-impact journals (Nature, FASEB J, Free Radic Biol Med, and Am J Physiol) [99,100,101,102]. Furthermore, studies published in high-impact journals (Figure 5A) and the bursts of keywords (expression, metabolism, and protein synthesis; Figure 7B) all focused on the morphological hallmarks of eccentric-contraction-induced injury and/or the protective effect of nutritional supplements, such as the myofibrillar Z-disc, α-actin, and the cytoskeleton (desmin, titin, nebulin, dystrophin, dysferlin, and so on) [8,67,103,104]. Since studies published in high-centrality journals such as Nature [101,102] revealed that the most common human muscular myopathy (Duchenne muscular dystrophy) is caused by a lack of dystrophin on the cytoplasmic surface of the skeletal muscle membrane, cytoskeleton proteins have been the focus. Furthermore, cytoskeleton degradation is an important potential mechanism of muscle damage/EIMD [8,67,103,104]. Accordingly, despite significant research contributions to EIMD, the precise mechanisms causing muscle damage, soreness, repair, and adaptation remain speculative, calling for further research.

### 4.4. Limitations

This bibliometric study had some limitations. Despite developing thorough and prudent search strategies and attempting to include as much of the literature published in various formats as possible, we only retrieved articles from the WoSCC database and included only English articles. The few articles that were not included in WoSCC and were published in languages other than English may have been overlooked [19,20]. However, full reference texts and citation lists are not available in most databases, such as Embase, MEDLINE, and Google Scholar. Thus, we could not cover all publications on this topic. As we all know, bibliometric research is primarily based on co-citations [18,19,20,21,26]. Because of the possibility of bias, our findings should be interpreted with at least some caution.

Because of the aforementioned potential biases, potential publications that are not adequately cited, publications and subsequent citations with a lag, and recent research trends, among others, may be difficult to detect. Moreover, the information we currently provide is based on selected material that focuses on the impact of protein and amino acids on EIMD. Although we explored the topics and frontiers of this field to the greatest extent possible using the results of co-citation reference networks and co-occurrence analysis, technical shortcomings may still exist. As a result, we believe that in the future, researchers should consider more detailed and targeted work in the following areas: (1) natural supplementation, plant versus animal proteins, or casein versus albumin in preventing or reversing the effects of EIMD; and (2) the timing and working period of protein, amino acid, and amino acid–carbohydrate ingestion.

## 5. Conclusions

This study provides, for the first time, a broad overview of existing EIMD research as well as useful insights into future research directions through bibliometric and visual analysis. The quantitative and qualitative results revealed 351 total publications from 1998 to 2022, the majority of which were concentrated in high-quality journals in the disciplines of Sport Science, Physiology, Nutrition, and Biochemistry & Molecular Biology. The top ten journals in the number of publications are mostly high-quality printed journals, and the top ten journals in centrality have an average impact factor of 13.845. The United States has the highest total number of publications. Notably, institutional cooperation is mostly geographically limited, with few transnational cooperation links.

By accessing areas of scientific knowledge and trends over time, we were able to determine that “exercise patterns”, “nutritional strategies”, “beneficial outcomes”, and “proposed mechanisms” were the most discussed research topics. Furthermore, we identified the following research frontiers and research directions: developing a comprehensive understanding of new exercise/training models, nutritional strategies, and recovery techniques to alleviate EIMD symptoms and accelerate recovery; applying the concept of hormesis in EIMD to induce muscle hypertrophy; and investigating the underlying mechanisms of muscle fiber and membrane damage.

## Figures and Tables

**Table 1:** The top ten journals of EIMD and protein intake studies ranked by number of publications.

Journal	Publications (%)	IF (2021)	H-Index	Country	Quartile in Category (2021)
Eur J Appl Physiol	20 (5.68%)	3.346	142	Germany	Physiology/Q2; Sport Sciences/Q2
Appl Physiol Nutr Metab	16 (4.46%)	3.016	95	Canada	Nutrition & Dietetic/Q3; Physiology/Q2; Sport Sciences/Q2
Med Sci Sports Exerc	13 (3.69%)	6.289	238	United States	Sport Sciences/Q1
J Strength Cond Res	12 (3.41%)	4.948	140	United States	Sport Sciences/Q1
J Int Soc Sports Nutr	12 (3.41%)	5.159	56	United Kingdom	Nutrition & Dietetics/Q2; Sport Sciences/Q1
J Appl Physiol	10 (2.84%)	3.880	240	United States	Physiology/Q2; Sport Sciences/Q2
Nutrients	10 (2.84%)	6.706	143	Switzerland	Nutrition & Dietetics/Q1
J Sport Sci	7 (1.99%)	3.943	145	United Kingdom	Sport Sciences/Q2
Eur J Sport Sci	6 (1.71%)	3.980	65	United Kingdom	Sport Sciences/Q2
Front Physiol	6 (1.71%)	4.755	122	Switzerland	Physiology/Q1

**Table 2:** The top ten EIMD and protein intake studies with the most citations.

Title	First Author (Country)	Journal	Citations	Acritical Type, Year of Publication
Exercise-induced muscle damage in humans [1]	Clarkson P.M. (USA)	Am J Phys Med Rehabil	840	Review, 2002
The prevention and treatment of exercise-induced muscle damage [10]	Howatson G. (UK)	Sports Med	302	Review, 2008
Eccentric exercise-induced injuries to contractile and cytoskeletal muscle fibre components [29]	Fridén J. (Sweden)	Acta Physiol Scand	268	Review, 2001
Time-course of changes in inflammatory and performance responses following a soccer game [30]	Ispirlidis I. (Greece)	Clin J Sport Med	243	Original article, 2008
Muscle damage and inflammation during recovery from exercise [31]	Peake J.M. (Australia)	J Appl Physiol	229	Review, 2017
Exercise promotes BCAA catabolism: Effects of BCAA supplementation on skeletal muscle during exercise [32]	Shimomura Y. (Japan)	J Nutr	213	Review, 2004
The cytokine response to strenuous exercise [27]	Pedersen B.K. (Denmark)	Can J Physiol Pharmacol	188	Review, 1998
Massage therapy attenuates inflammatory signaling after exercise-induced muscle damage [16]	Crane J.D. (Canada)	Sci Transl Med	168	Original article, 2012
Nutraceutical effects of branched-chain amino acids on skeletal muscle [35]	Shimomura Y. (Japan)	J Nutr	159	Original article, 2006
Influence of cold-water immersion on indices of muscle damage following prolonged intermittent shuttle running [17]	Bailey D.M. (UK)	J Sports Sci	143	Original article, 2007

Note: Article type = original article or review.

**Table 3:** The top ten EIMD and protein intake studies with the highest centrality.

Title	First Author	Journal	Centrality	Acritical Type, Year of Publication
Effect of milk-based carbohydrate-protein supplement timing on the attenuation of exercise-induced muscle damage [33]	Cockburn E. (UK)	Appl Physiol Nutr Metab	0.29	Original article, 2010
The prevention and treatment of exercise-induced muscle damage [10]	Howatson G. (UK)	Sports Med	0.23	Review, 2008
Acute milk-based protein-CHO supplementation attenuates exercise-induced muscle damage [34]	Cockburn E. (UK)	Appl Physiol Nutr Metab	0.20	Original article, 2008
Montmorency cherries reduce the oxidative stress and inflammatory responses to repeated days high-intensity stochastic cycling [36]	Bell P.G. (UK)	Nutrients	0.19	Original article, 2014
Dietary strategies to recover from exercise-induced muscle damage [11]	Sousa M. (Portugal)	Int J Food Sci Nutr	0.16	Review, 2014
Delayed onset muscle soreness: treatment strategies and performance factors [5]	Cheung K. (New Zealand)	Sports Med	0.15	Review, 2003
Exercise-induced muscle damage: What is it, what causes it and what are the nutritional solutions? [6]	Owens D.J. (UK)	Eur J Sport Sci	0.14	Review, 2019
Effects of dietary carbohydrate on delayed onset muscle soreness and reactive oxygen species after contraction induced muscle damage [37]	Close G.L. (UK)	Br J Sports Med	0.13	Original article, 2005
Effects of a carbohydrate-protein beverage on cycling endurance and muscle damage [38]	Saunders M.J. (USA)	Med Sci Sport Exer	0.13	Original article, 2004
Does exercise-induced muscle damage play a role in skeletal muscle hypertrophy? [14]	Schoenfeld B.J. (USA)	J Strength Cond Res	0.13	Review, 2012

Note: Article type = original article or review.

## Data Availability

The data presented in this study are available in this manuscript.

## Supplementary Materials

The following supporting information can be downloaded at: https://www.mdpi.com/article/10.3390/nu14204288/s1. Table S1: Search strategy and results, Table S2: Key concepts in bibliometrics and in network analysis.

## References

[1] Clarkson P.M., Hubal M.J. (2002). Exercise-induced muscle damage in humans. Am. J. Phys. Med. Rehabil..

[2] Clarkson P.M., Nosaka K., Braun B. (1992). Muscle function after exercise-induced muscle damage and rapid adaptation. Med. Sci. Sports Exerc..

[3] Brown S., Day S., Donnelly A. (1999). Indirect evidence of human skeletal muscle damage and collagen breakdown after eccentric muscle actions. J. Sports Sci..

[4] Peake J.M., Roberts L.A., Figueiredo V.C., Egner I., Krog S., Aas S.N., Suzuki K., Markworth J.F., Coombes J.S., Cameron-Smith D. (2017). The effects of cold water immersion and active recovery on inflammation and cell stress responses in human skeletal muscle after resistance exercise. J. Physiol..

[5] Cheung K., Hume P., Maxwell L. (2003). Delayed onset muscle soreness: Treatment strategies and performance factors. Sports Med..

[6] Owens D.J., Twist C., Cobley J.N., Howatson G., Close G.L. (2019). Exercise-induced muscle damage: What is it, what causes it and what are the nutritional solutions?. Eur. J. Sport Sci..

[7] Hyldahl R.D., Hubal M.J. (2014). Lengthening our perspective: Morphological, cellular, and molecular responses to eccentric exercise. Muscle Nerve.

[8] Yu J.G., Liu J.X., Carlsson L., Thornell L.E., Stal P.S. (2013). Re-evaluation of sarcolemma injury and muscle swelling in human skeletal muscles after eccentric exercise. PLoS ONE.

[9] McHugh M.P. (2003). Recent advances in the understanding of the repeated bout effect: The protective effect against muscle damage from a single bout of eccentric exercise. Scand. J. Med. Sci. Sports.

[10] Howatson G., van Someren K.A. (2008). The prevention and treatment of exercise-induced muscle damage. Sports Med..

[11] Sousa M., Teixeira V.H., Soares J. (2014). Dietary strategies to recover from exercise-induced muscle damage. Int. J. Food Sci. Nutr..

[12] Bongiovanni T., Genovesi F., Nemmer M., Carling C., Alberti G., Howatson G. (2020). Nutritional interventions for reducing the signs and symptoms of exercise-induced muscle damage and accelerate recovery in athletes: Current knowledge, practical application and future perspectives. Eur. J. Appl. Physiol..

[13] Markus I., Constantini K., Hoffman J.R., Bartolomei S., Gepner Y. (2021). Exercise-induced muscle damage: Mechanism, assessment and nutritional factors to accelerate recovery. Eur. J. Appl. Physiol..

[14] Schoenfeld B.J. (2012). Does exercise-induced muscle damage play a role in skeletal muscle hypertrophy?. J. Strength Cond Res..

[15] Baumert P., Lake M.J., Stewart C.E., Drust B., Erskine R.M. (2016). Genetic variation and exercise-induced muscle damage: Implications for athletic performance, injury and ageing. Eur. J. Appl. Physiol..

[16] Crane J.D., Ogborn D.I., Cupido C., Melov S., Hubbard A., Bourgeois J.M., Tarnopolsky M.A. (2012). Massage therapy attenuates inflammatory signaling after exercise-induced muscle damage. Sci. Transl. Med..

[17] Bailey D.M., Erith S.J., Griffin P.J., Dowson A., Brewer D.S., Gant N., Williams C. (2007). Influence of cold-water immersion on indices of muscle damage following prolonged intermittent shuttle running. J. Sports Sci..

[18] Nakagawa S., Samarasinghe G., Haddaway N.R., Westgate M.J., O’Dea R.E., Noble D.W.A., Lagisz M. (2019). Research weaving: Visualizing the future of research synthesis. Trends Ecol. Evol..

[19] Chen J., Xue X., Xu J., Zeng J., Xu F. (2022). Emerging trends and hotspots in tai chi fall prevention: Analysis and visualization. Int. J. Environ. Res. Public Health.

[20] Xu F., Xu J., Zhou D., Xie H., Liu X. (2022). A bibliometric and visualization analysis of motor learning in preschoolers and children over the last 15 years. Healthcare.

[21] Cortese S., Sabe M., Chen C., Perroud N., Solmi M. (2022). Half a century of research on attention-deficit/hyperactivity disorder: A scientometric study. Neurosci. Biobehav. Rev..

[22] Hirsch J.E. (2005). An index to quantify an individual’s scientific research output. Proc. Natl Acad Sci. USA.

[23] Small H. (1973). Co-citation in the scientific literature: A new measure of the relationship between two documents. J. Am. Soc. Inf. Sci..

[24] Hofmann T., Puzicha J. (1998). Statistical Models for Co-Occurrence Data.

[25] White H.D., McCain K.W. (1998). Visualizing a discipline: An author co-citation analysis of information science, 1972–1995. J. Am. Soc. Inf. Sci..

[26] Chen C. (2004). Searching for intellectual turning points: Progressive knowledge domain visualization. Proc. Natl. Acad. Sci. USA.

[27] Pedersen B.K., Ostrowski K., Rohde T., Bruunsgaard H. (1998). The cytokine response to strenuous exercise. Can. J. Physiol. Pharmacol..

[28] Bradford M.M. (1976). A rapid and sensitive method for the quantitation of microgram quantities of protein utilizing the principle of protein-dye binding. Anal. Biochem..

[29] Friden J., Lieber R.L. (2001). Eccentric exercise-induced injuries to contractile and cytoskeletal muscle fibre components. Acta Physiol. Scand..

[30] Ispirlidis I., Fatouros I.G., Jamurtas A.Z., Nikolaidis M.G., Michailidis I., Douroudos I., Margonis K., Chatzinikolaou A., Kalistratos E., Katrabasas I. (2008). Time-course of changes in inflammatory and performance responses following a soccer game. Clin. J. Sport Med..

[31] Peake J.M., Neubauer O., Della Gatta P.A., Nosaka K. (2017). Muscle damage and inflammation during recovery from exercise. J. Appl. Physiol..

[32] Shimomura Y., Murakami T., Nakai N., Nagasaki M., Harris R.A. (2004). Exercise promotes bcaa catabolism: Effects of BCAA supplementation on skeletal muscle during exercise. J. Nutr..

[33] Cockburn E., Stevenson E., Hayes P.R., Robson-Ansley P., Howatson G. (2010). Effect of milk-based carbohydrate-protein supplement timing on the attenuation of exercise-induced muscle damage. Appl. Physiol. Nutr. Metab..

[34] Cockburn E., Hayes P.R., French D.N., Stevenson E., St Clair Gibson A. (2008). Acute milk-based protein-cho supplementation attenuates exercise-induced muscle damage. Appl. Physiol. Nutr. Metab..

[35] Shimomura Y., Yamamoto Y., Bajotto G., Sato J., Murakami T., Shimomura N., Kobayashi H., Mawatari K. (2006). Nutraceutical effects of branched-chain amino acids on skeletal muscle. J. Nutr..

[36] Bell P.G., Walshe I.H., Davison G.W., Stevenson E., Howatson G. (2014). Montmorency cherries reduce the oxidative stress and inflammatory responses to repeated days high-intensity stochastic cycling. Nutrients.

[37] Close G.L., Ashton T., Cable T., Doran D., Noyes C., McArdle F., MacLaren D.P. (2005). Effects of dietary carbohydrate on delayed onset muscle soreness and reactive oxygen species after contraction induced muscle damage. Br. J. Sports Med..

[38] Saunders M.J., Kane M.D., Todd M.K. (2004). Effects of a carbohydrate-protein beverage on cycling endurance and muscle damage. Med. Sci. Sports Exerc..

[39] Harty P.S., Cottet M.L., Malloy J.K., Kerksick C.M. (2019). Nutritional and supplementation strategies to prevent and attenuate exercise-induced muscle damage: A brief review. Sports Med. Open.

[40] Fatouros I.G., Jamurtas A.Z. (2016). Insights into the molecular etiology of exercise-induced inflammation: Opportunities for optimizing performance. J Inflamm. Res.

[41] Bell P.G., Stevenson E., Davison G.W., Howatson G. (2016). The effects of montmorency tart cherry concentrate supplementation on recovery following prolonged, intermittent exercise. Nutrients.

[42] Damas F., Nosaka K., Libardi C.A., Chen T.C., Ugrinowitsch C. (2016). Susceptibility to exercise-induced muscle damage: A cluster analysis with a large sample. Int. J. Sports Med..

[43] Bell P.G., Walshe I.H., Davison G.W., Stevenson E.J., Howatson G. (2015). Recovery facilitation with montmorency cherries following high-intensity, metabolically challenging exercise. Appl. Physiol. Nutr. Metab..

[44] Yu J.G., Carlsson L., Thornell L.E. (2004). Evidence for myofibril remodeling as opposed to myofibril damage in human muscles with DOMS: An ultrastructural and immunoelectron microscopic study. Histochem Cell Biol..

[45] Yuan Z.M., Li M., Ji C.Y., Li L., Jia L., Incecik A. (2019). Steady hydrodynamic interaction between human swimmers. J. R. Soc. Interface.

[46] Twist C., Eston R. (2005). The effects of exercise-induced muscle damage on maximal intensity intermittent exercise performance. Eur. J. Appl. Physiol..

[47] Baty J.J., Hwang H., Ding Z., Bernard J.R., Wang B., Kwon B., Ivy J.L. (2007). The effect of a carbohydrate and protein supplement on resistance exercise performance, hormonal response, and muscle damage. J. Strength Cond. Res..

[48] White J.P., Wilson J.M., Austin K.G., Greer B.K., St John N., Panton L.B. (2008). Effect of carbohydrate-protein supplement timing on acute exercise-induced muscle damage. J. Int. Soc. Sports Nutr..

[49] Betts J.A., Toone R.J., Stokes K.A., Thompson D. (2009). Systemic indices of skeletal muscle damage and recovery of muscle function after exercise: Effect of combined carbohydrate-protein ingestion. Appl. Physiol. Nutr. Metab..

[50] Green M.S., Corona B.T., Doyle J.A., Ingalls C.P. (2008). Carbohydrate-protein drinks do not enhance recovery from exercise-induced muscle injury. Int. J. Sport Nutr. Exerc. Metab..

[51] Howatson G., McHugh M.P., Hill J.A., Brouner J., Jewell A.P., van Someren K.A., Shave R.E., Howatson S.A. (2010). Influence of tart cherry juice on indices of recovery following marathon running. Scand. J. Med. Sci. Sports.

[52] Choi J.H., Jin S.W., Kim H.G., Choi C.Y., Lee H.S., Ryu S.Y., Chung Y.C., Hwang Y.J., Um Y.J., Jeong T.C. (2015). Saponins, especially platyconic acid A, from platycodon grandiflorum reduce airway inflammation in ovalbumin-induced mice and PMA-exposed A549 cells. J. Agric. Food Chem..

[53] Hutchison A.T., Flieller E.B., Dillon K.J., Leverett B.D. (2016). Black currant nectar reduces muscle damage and inflammation following a bout of high-intensity eccentric contractions. J. Diet. Suppl..

[54] Machado A.F., Ferreira P.H., Micheletti J.K., de Almeida A.C., Lemes I.R., Vanderlei F.M., Netto Junior J., Pastre C.M. (2016). Can water temperature and immersion time influence the effect of cold water immersion on muscle soreness? A systematic review and meta-analysis. Sports Med..

[55] Kanda K., Sugama K., Hayashida H., Sakuma J., Kawakami Y., Miura S., Yoshioka H., Mori Y., Suzuki K. (2013). Eccentric exercise-induced delayed-onset muscle soreness and changes in markers of muscle damage and inflammation. Exerc. Immunol. Rev..

[56] Xiao K., Chen Y., Jiang X., Tyagi V.K., Zhou Y. (2016). Characterization of key organic compounds affecting sludge dewaterability during ultrasonication and acidification treatments. Water Res..

[57] Clark K.A., McElhinny A.S., Beckerle M.C., Gregorio C.C. (2002). Striated muscle cytoarchitecture: An intricate web of form and function. Annu. Rev. Cell Dev. Biol..

[58] Howatson G., Hoad M., Goodall S., Tallent J., Bell P.G., French D.N. (2012). Exercise-induced muscle damage is reduced in resistance-trained males by branched chain amino acids: A randomized, double-blind, placebo controlled study. J. Int. Soc. Sports Nutr..

[59] Greer B.K., Woodard J.L., White J.P., Arguello E.M., Haymes E.M. (2007). Branched-chain amino acid supplementation and indicators of muscle damage after endurance exercise. Int. J. Sport Nutr. Exerc. Metab..

[60] Jackman S.R., Witard O.C., Jeukendrup A.E., Tipton K.D. (2010). Branched-chain amino acid ingestion can ameliorate soreness from eccentric exercise. Med. Sci. Sports Exerc..

[61] Ascensao A., Rebelo A., Oliveira E., Marques F., Pereira L., Magalhaes J. (2008). Biochemical impact of a soccer match—Analysis of oxidative stress and muscle damage markers throughout recovery. Clin. Biochem..

[62] Baird M.F., Graham S.M., Baker J.S., Bickerstaff G.F. (2012). Creatine-kinase- and exercise-related muscle damage implications for muscle performance and recovery. J. Nutr. Metab..

[63] Nikolaidis M.G., Jamurtas A.Z., Paschalis V., Fatouros I.G., Koutedakis Y., Kouretas D. (2008). The effect of muscle-damaging exercise on blood and skeletal muscle oxidative stress: Magnitude and time-course considerations. Sports Med..

[64] Nosaka K., Sacco P., Mawatari K. (2006). Effects of amino acid supplementation on muscle soreness and damage. Int. J. Sport Nutr. Exerc. Metab..

[65] Tipton K.D., Elliott T.A., Cree M.G., Aarsland A.A., Sanford A.P., Wolfe R.R. (2007). Stimulation of net muscle protein synthesis by whey protein ingestion before and after exercise. Am. J. Physiol. Endocrinol. Metab..

[66] Clifford T., Bell O., West D.J., Howatson G., Stevenson E.J. (2016). The effects of beetroot juice supplementation on indices of muscle damage following eccentric exercise. Eur. J. Appl. Physiol..

[67] Yu J.G., Furst D.O., Thornell L.E. (2003). The mode of myofibril remodelling in human skeletal muscle affected by DOMS induced by eccentric contractions. Histochem. Cell Biol..

[68] Saltin B., Grimby G. (1968). Physiological analysis of middle-aged and old former athletes. Comparison with still active athletes of the same ages. Circulation.

[69] Saltin B., Helge J.W. (2000). Metabolic capacity of skeletal muscles and health. Ugeskr. Laeger..

[70] Bangsbo J., Graham T.E., Kiens B., Saltin B. (1992). Elevated muscle glycogen and anaerobic energy production during exhaustive exercise in man. J. Physiol..

[71] Bangsbo J., Johansen L., Graham T., Saltin B. (1993). Lactate and H^+^ effluxes from human skeletal muscles during intense, dynamic exercise. J. Physiol..

[72] Bangsbo J., Johansen L., Quistorff B., Saltin B. (1993). NMR and analytic biochemical evaluation of CRP and nucleotides in the human calf during muscle contraction. J. Appl. Physiol..

[73] Friden J., Lieber R.L., Thornell L.E. (1991). Subtle indications of muscle damage following eccentric contractions. Acta Physiol. Scand..

[74] Malm C. (2001). Exercise-induced muscle damage and inflammation: Fact or fiction?. Acta Physiol. Scand..

[75] Malm C., Yu J.G. (2012). Exercise-induced muscle damage and inflammation: Re-evaluation by proteomics. Histochem. Cell Biol..

[76] Vihko V., Salminen A., Rantamaki J. (1979). Exhaustive exercise, endurance training, and acid hydrolase activity in skeletal muscle. J. Appl. Physiol. Respir. Environ. Exerc. Physiol..

[77] Vihko V., Salminen A. (1979). Damage of skeletal muscle tissue during physical stress. Duodecim.

[78] Schwane J.A., Watrous B.G., Johnson S.R., Armstrong R.B. (1983). Is lactic acid related to delayed-onset muscle soreness?. Phys. Sportsmed..

[79] Stedge H.L., Armstrong K. (2021). The effects of intermittent pneumatic compression on the reduction of exercise-induced muscle damage in endurance athletes: A critically appraised topic. J. Sport Rehabil..

[80] Schwane J.A., Johnson S.R., Vandenakker C.B., Armstrong R.B. (1983). Delayed-onset muscular soreness and plasma CPK and LDH activities after downhill running. Med. Sci. Sports Exerc..

[81] Millet G.Y., Tomazin K., Verges S., Vincent C., Bonnefoy R., Boisson R.C., Gergele L., Feasson L., Martin V. (2011). Neuromuscular consequences of an extreme mountain ultra-marathon. PLoS ONE.

[82] Nosaka K., Newton M., Sacco P. (2002). Delayed-onset muscle soreness does not reflect the magnitude of eccentric exercise-induced muscle damage. Scand. J. Med. Sci. Sports.

[83] Leeder J.D., van Someren K.A., Gaze D., Jewell A., Deshmukh N.I., Shah I., Barker J., Howatson G. (2014). Recovery and adaptation from repeated intermittent-sprint. exercise. Int. J. Sports Physiol. Perform..

[84] Chen T.C., Nosaka K., Lin M.J., Chen H.L., Wu C.J. (2009). Changes in running economy at different intensities following downhill running. J. Sports Sci..

[85] Mielgo-Ayuso J., Calleja-Gonzalez J., Del Coso J., Urdampilleta A., Leon-Guereno P., Fernandez-Lazaro D. (2019). Caffeine supplementation and physical performance, muscle damage and perception of fatigue in soccer players: A systematic review. Nutrients.

[86] Koyama T., Rikukawa A., Nagano Y., Sasaki S., Ichikawa H., Hirose N. (2022). High-acceleration movement, muscle damage, and perceived exertion in basketball games. Int. J. Sports Physiol. Perform..

[87] Simmons R., Doma K., Sinclair W., Connor J., Leicht A. (2021). Acute effects of training loads on muscle damage markers and performance in semi-elite and elite athletes: A systematic review and meta-analysis. Sports Med..

[88] Hyldahl R.D., Chen T.C., Nosaka K. (2017). Mechanisms and mediators of the skeletal muscle repeated bout effect. Exerc. Sport Sci. Rev..

[89] Jones L., Bailey S.J., Rowland S.N., Alsharif N., Shannon O.M., Clifford T. (2021). The effect of nitrate-rich beetroot juice on markers of exercise-induced muscle damage: A systematic review and meta-analysis of human intervention trials. J. Diet. Suppl..

[90] Beelen M., Burke L.M., Gibala M.J., van Loon L.J. (2010). Nutritional strategies to promote postexercise recovery. Int. J. Sport Nutr. Exerc. Metab..

[91] Slater G.J., Jenkins D. (2000). Beta-hydroxy-beta-methylbutyrate (HMB) supplementation and the promotion of muscle growth and strength. Sports Med..

[92] Kim Y.A., Oh S.H., Lee G.H., Hoa P.T., Jin S.W., Chung Y.C., Lee Y.C., Jeong H.G. (2018). Platycodon grandiflorum-derived saponin attenuates the eccentric exercise-induced muscle damage. Food Chem. Toxicol..

[93] Burke L.M., Castell L.M., Stear S.J. (2009). Bjsm reviews: A-z of supplements: Dietary supplements, sports nutrition foods and ergogenic aids for health and performance part 1. Br. J. Sports Med..

[94] Hunter G.R., McCarthy J.P., Bamman M.M. (2004). Effects of resistance training on older adults. Sports Med..

[95] LaStayo P., McDonagh P., Lipovic D., Napoles P., Bartholomew A., Esser K., Lindstedt S. (2007). Elderly patients and high force resistance exercise—A descriptive report: Can an anabolic, muscle growth response occur without muscle damage or inflammation?. J. Geriatr. Phys. Ther..

[96] Flann K.L., LaStayo P.C., McClain D.A., Hazel M., Lindstedt S.L. (2011). Muscle damage and muscle remodeling: No pain, no gain?. J. Exp. Biol..

[97] Radak Z., Chung H.Y., Goto S. (2005). Exercise and hormesis: Oxidative stress-related adaptation for successful aging. Biogerontology.

[98] Radak Z., Chung H.Y., Koltai E., Taylor A.W., Goto S. (2008). Exercise, oxidative stress and hormesis. Ageing Res. Rev..

[99] Hoffman E.P., Brown R.H., Kunkel L.M. (1987). Dystrophin: The protein product of the duchenne muscular dystrophy locus. Cell.

[100] Koenig M., Monaco A.P., Kunkel L.M. (1988). The complete sequence of dystrophin predicts a rod-shaped cytoskeletal protein. Cell.

[101] Watkins S.C., Hoffman E.P., Slayter H.S., Kunkel L.M. (1988). Immunoelectron microscopic localization of dystrophin in myofibres. Nature.

[102] Bansal D., Miyake K., Vogel S.S., Groh S., Chen C.C., Williamson R., McNeil P.L., Campbell K.P. (2003). Defective membrane repair in dysferlin-deficient muscular dystrophy. Nature.

[103] Xu F., Huang Q.T., Cao J.M., Wang P., Xu Y.M. (2017). Exploration and verification of MAPK mechanism of hypoxia regulates sarcolemma injury after EIMD. China Sport Sci..

[104] Xu F., Qin L.F., Huang Q.T., Cao J.M., Wang P., Xu Y.M. (2019). Hypoxia regulates calpain activity and the possible mechanism of sarcolemma injury after EIMD. China Sports Sci. Tech..

