# Supplementary material for: Exercise-Induced Muscle Damage and Protein Intake: A Bibliometric and Visual Analysis
Source: Nutrients. 2022 Oct 14;14(20):4288. doi: 10.3390/nu14204288 (PMC9610071; doi:10.3390/nu14204288)
Supplement: Supplementary file 1 [file nutrients-14-04288-s001.zip › nutrients-1890716-supplementary.pdf]

Table S1. Search strategy and results

Set	Results	*WoSCC* Search Strategies
#1	1,019	TOPIC = ("DOMS")
#2	858	TOPIC = ("delayed onset muscle soreness")
#3	244	TOPIC = ("EIMD")
#4	803	TOPIC = ("exercise-induced muscle damage")
#5	2,819,535	TOPIC = ("protein*")
#6	7,035	TOPIC = ("protein* intake")
#7	407	TOPIC = ("protein* ingestion")
#8	2043	#1 OR #2 OR #3 OR #4
#9	2,593,838	#5 OR #6 OR #7
#10	351	#6 AND #5

Note: Indexes (SCI-Expanded; SSCI; A&HCI; CPCI-S; CPCI-SSH; ESCI; CCR-Expanded; IC); Language (English); Timespan (1975–2022).

Table S2. Key concepts in bibliometrics and in network analysis

Term	Explanation
Key concepts	
Bibliometric	It enables researchers to see how pieces of evidence are connected, revealing the structure and development of a field
Systematic mapping	It provides a snapshot of the current state of knowledge in the scientific literature, identifying areas needing more research attention and those ready for full synthesis
Intellectual structure	It refers to a set of salient attributes of the knowledge base that can provide an organized and holistic understanding of the chosen scientific domain
Performance analysis	It quantifies citation impacts and productivity using several metrics, such as publication-related metrics (total publication, number of co-authors.), citation-related metrics (total citation, average citation), and citation and publication -related metrics (g-index, number of cited papers)
Collaboration networks	They are defined as a set of individuals who come together and collaborate on particular tasks such as publishing a paper
Key metrics	
Betweenness centrality	The algorithm calculates unweighted shortest paths between all pairs of nodes in a graph. Each node receives a score, based on the number of shortest paths that pass through the node
Burstness analysis	Citation burst is a detection of a burst event. A citation burst provides evidence that a particular<br>publication is associated with a surge of citations. Furthermore, if a cluster contains numerous nodes with strong<br>citation bursts, then the cluster as a whole capture an active area of research, or an emerging trend.
Modularity scores	Modularity is a measure of the structure of a graph, measuring the density of connections within a module. Graphs with a high modularity score will have many connections within a module but only few pointing outwards to other modules. Its value ranges from 0 to 1.
Silhouette scores	Silhouette score is a metric used to calculate the goodness of a clustering technique. Its value ranges from − 1 to 1.<br>1: clusters are isolated from each other.<br>0: clusters are indifferent, or we can say that the distance between clusters is not significant.<br>-1: clusters are assigned in the wrong way.
Centrality divergence	The centrality divergence metric measures the structural variation caused by an article a in terms of the divergence of the distribution of betweenness centrality CB of nodes vi in the baseline network. The centrality divergence metric is potentially valuable for detecting boundary-spanning activities at interdisciplinary levels.
Modularity divergence	The modularity of a network is a measure of the overall structure of the network. Its range is between − 1 and 1. The<br>modularity change rate of a scientific paper measures the relative structural change due to the information from the<br>published paper with reference to a baseline network.
